# Karyotypic and molecular evidence supports the endemic Tibetan hamsters as a separate divergent lineage of Cricetinae

**DOI:** 10.1038/s41598-021-89890-1

**Published:** 2021-05-18

**Authors:** Svetlana A. Romanenko, Vladimir S. Lebedev, Anna A. Bannikova, Svetlana V. Pavlova, Natalia A. Serdyukova, Natalia Yu. Feoktistova, Qu Jiapeng, Sun Yuehua, Alexey V. Surov, Alexander S. Graphodatsky

**Affiliations:** 1 Institute of Molecular and Cellular Biology (IMCB), Siberian Branch of Russian Academy of Sciences (SB RAS), 8/2 Lavrentjev Ave., 630090 Novosibirsk, Russia; 2 Zoological Museum of Moscow State University, 125009 Moscow, Russia; 3 Lomonosov Moscow State University, Vorobievy Gory, 119991 Moscow, Russia; 4 A.N. Severtsov Institute of Ecology and Evolution, RAS, 119071 Moscow, Russia; 5 Northwest Institute of Plateau Biology, Chinese Academy of Sciences, Xining, 810001 Qinghai Province People’s Republic of China; 6 Institute of Zoology, Chinese Academy of Sciences, Beijing, 100101 People’s Republic of China

**Keywords:** Cytogenetics, Evolutionary biology, Comparative genomics

## Abstract

The genus status of *Urocricetus* was defined recently based on morphological and molecular data. Even though the amount of evidence for a separate phylogenetic position of this genus among Cricetinae continues to increase, there is still no consensus on its relationship to other groups. Here we give the first comprehensive description of the *U. kamensis* karyotype (2*n* = 30, NF*a* = 50) including results of comparative cytogenetic analysis and detailed examination of its phylogenetic position by means of numerous molecular markers. The molecular data strongly indicated that *Urocricetus* is a distant sister group to *Phodopus*. Comparative cytogenetic data showed significant reorganization of the *U. kamensis* karyotype compared to karyotypes of all other hamsters investigated earlier. The totality of findings undoubtedly means that *Urocricetus* belongs to a separate divergent lineage of Cricetinae.

## Introduction

The Kam hamster *Urocricetus kamensis* (Satunin, 1903 [imprint 1902]) is a member of the lineage commonly known as Tibetan hamsters, which is one of the few rodent taxa endemic to the Qinghai-Tibet plateau where it is found at altitudes up to 5200 m above the sea level^1^. Over the past century, the classification of Tibetan hamsters based on morphological characteristics has been controversial^2, 3^. Superficial similarity with another group of small hamsters affiliated with the genus *Cricetulus* Milne-Edwards, 1867 (e.g. *C. barabensis* (Pallas, 1773); *C. longicaudatus* (Milne-Edwards, 1867)) has led to the situation where Tibetan hamsters have been traditionally included within the latter genus, often as the subgenus *Urocricetus* (Satunin, 1903 [imprint 1902]).

The first available mitochondrial data suggested that the Tibetan-hamster lineage is unrelated to all other *Cricetulus* members^4, 5^. Subsequently, this conclusion has been corroborated by phylogenetic reconstructions using a set of five nuclear exons and two mitochondrial genes^2^. Furthermore, molecular evidence has indicated that *Urocricetus* is phylogenetically related to dwarf hamsters of the genus *Phodopus* (Miller, 1910)^2, 6^. It was suggested that both *Phodopus* and *Urocricetus* originated in East Central Asia (east of the Altai–Tian Shan boundary) having separated from each other in the early Late Miocene (~ 10.4 Mya), which is comparable to the time point of the split between chromosomally, morphologically, and genetically divergent *Mesocricetus* and *Cricetus* lineages. Accordingly, it has been postulated that *Urocricetus* definitely deserves the rank of a separate genus^2^.

Species structure of this genus is unclear. At different times, one to four species of Tibetan hamsters were believed to exit by different authors (e.g., ref.^1, 7^; for more details see ref.^3^). Nonetheless, in most checklists, only *U. kamensis* and *U. alticola* (Thomas, 1917) have been regarded as valid species (e.g., ref.^8^), as *Cricetulus* members. Recent nuclear and mitochondrial data are consistent with the view that *U. kamensis* and *U. alticola* are distinct, albeit closely related species^2^; however, this treatment is opposed by Ding and Liao^3^, who advocate the monotypy of the genus.

Available molecular data on *Urocricetus* are limited to sequences of mitochondrial DNA and several exons of nuclear genes. There is absolutely no information on karyotypic organization of genomes of its representatives. In this work, for the first time, we give a complete description of *U. kamensis* karyotype structure. To precisely determine the phylogenetic position of *Urocricetus*, we undertook a sequence analysis of fragments of 25 nuclear genes.

## Materials and methods

### Compliance with ethical standards

The study was carried out in compliance with the ARRIVE guidelines. All experiments were approved by the Ethics Committee on Animal and Human Research at the IMCB SB RAS, Russia (protocol No. 01/20 of 11 February 2020), following all relevant guidelines and regulations. This article does not contain any experiments on human subjects performed by any of the coauthors.

### Specimens sampled

The Kam hamsters used in the study were collected in July 2018 in Beizha area, Nangqen county, Qinghai province, China (N31°52′56′′, E96°33′16.2′′ and N31°53′37′′, E96°35′55′′). This site is close to the terra typica of *U. kamensis* (Satunin, 1903 [imprint 1902]), which was designated originally as “River Moktschjun, district of Mekong, North-Eastern Tibet.” Each collected specimen received its personal field number (see Supplementary Information). One male (Q18-81) and one female (Q18-96) were karyotyped, and Q18-96 was subjected to a molecular cytogenetic analysis. Mitochondrial cytochrome *b* gene was sequenced in two specimens (Q18-82 and Q18-90), and for specimen Q18-90, the fragments of 25 nuclear DNA loci were sequenced.

### Chromosome preparation and staining

Mitotic chromosome suspensions of a *U. kamensis* male were obtained by the standard mitotic technique from short-term culture of bone marrow after colchicine treatment in vivo following the general protocol^9^. Only a *U. kamensis* female was used for the molecular cytogenetic analysis; metaphase chromosome spreads were prepared from primary fibroblast cultures as described previously^10, 11^. The fibroblastic cell lines were derived from biopsies of lung, rib, and tail tissues. All the cell lines were deposited in the IMCB cell bank (The General Collection of Cell Cultures, No. 0310–2016-0002). Cell lines of *Phodopus roborovskii*, *Phodopus sungorus*, and *Mesocricetus auratus* (the golden hamster) were retrieved from the same cell bank. All cell cultures and chromosome suspensions from them were obtained in the Laboratory of Animal Cytogenetics, IMCB SB RAS, Novosibirsk, Russia.

G-banding was performed by the standard trypsin/Giemsa procedure^12^. C-banding was carried out following the classic method or a previously published technique^13^ with some modifications^14^. CDAG-banding was conducted as described before^15^.

### Microdissection, probe amplification, and labeling

Glass needle–based microdissection was performed on G-banded chromosomes as described earlier^16^. One copy of each chromosome was collected. Chromosome-specific libraries were created with whole-genome amplification kits (Sigma). After the amplification, DNA was purified by means of nucleic acid purification kits for DNA (BioSilica). DNA libraries were labeled by whole-genome amplification kits (Sigma) according to the manufacturer's protocol. Chromosome-specific probes were created for both homologous chromosomes 13 and homologous chromosomes 14 of the female *U. kamensis* (Q18-96) and for chromosomes and chromosome regions of *M. auratus* (MAUR): 6q distal, 6q proximal, 9, 11, 13, 14, 15, and 18 distal.

### Fluorescence in situ hybridization (FISH)

The set of chromosome-specific probes and some microdissected painting probes of the golden hamster *M. auratus* (2n = 44) were described earlier^17^. The telomeric DNA probe was generated by PCR with oligonucleotides (TTAGGG)_5_ and (CCCTAA)_5_^18^. Clones of human ribosomal DNA (rDNA) containing a partial 18S ribosomal gene, the full 5.8S gene, a part of the 28S gene, and two internal transcribed spacers were obtained as described elsewhere^19^. FISH was performed in accordance with previously published protocols. Images were captured using the VideoTest-FISH software (Imicrotec) with a JenOptic charge-coupled device (CCD) camera mounted on an Olympus BX53 microscope. Hybridization signals were assigned to specific chromosome regions identified by means of G-banding patterns photographed by the CCD camera. All images were processed in Corel Paint Shop Pro X3 (Jasc Software).

### Molecular data

Molecular procedures employed for the sequencing of 25 nuclear genes and the mitochondrial *Cytb* gene are described in detail in Supplementary Information. The nuclear dataset included GenBank sequences of three other Old World hamster species (*Cricetulus [barabensis] griseus*, *Mesocricetus auratus*, and *Phodopus ex gr. sungorus*), which, along with *Urocricetus*, represent the main four lineages of Cricetinae according to ref.^2^. The composite outgroup included members of other cricetid subfamilies: *Peromyscus* sp*.* (Neotominae), *Sigmodon* sp*.* (Sigmodontinae), *Ondatra zibethica*, and *Ellobius talpinus* (Arvicolinae). The sequences were aligned by eye in BioEdit version 7.0.9.0^20^ and then trimmed to exclude poorly alignable intronic regions. The length of the final nuclear alignment was 13,125 bp.

### Phylogenetic analysis

Phylogenetic trees were reconstructed based on nuclear concatenation under maximum likelihood (ML) and Bayesian criteria. The ML reconstructions were conducted in IQTree version 1.6^21^. The ModelFinder routine^22^ was utilized to determine the optimal partitioning scheme and best-fit substitution models for each subset under the Bayesian information criterion. At the initial stage, all gene × codon position combinations were treated as independent subsets. We tested both edge-linked proportional and edge-unlinked partition models. Clade support was assessed using Ultrafast Bootstrap^23^ with 10,000 replicates and by aBayes and SH-aLRT tests^24^; the latter procedure was carried out with 10,000 bootstrap replicates.

To identify a confidence set of trees, we calculated probabilities of all 15 possible topologies for Cricetinae by the approximately-unbiased (AU) test^25^. The branching pattern of outgroups was fixed to that in the ML tree. The test was performed in IQTree with 100,000 bootstrap replicates.

For single-gene analyses, ML trees were inferred as described above for each of the 25 genes. Gene tree concordance was evaluated by the AU tests contrasting the ML gene-specific topology with the ML tree for concatenated data.

Bayesian tree reconstruction and node age estimation were conducted in BEAST ver. 1.10.4^26^ via concatenated alignment. The partitioning scheme and substitution models were those inferred by the ML procedure under the edge-unlinked partition model. Separate clock models were used for each data subset. Taking into account that the pattern of rate variation is unknown, we applied two relaxed-clock models: the uncorrelated log-normal clock and random local clock. Diffuse uniform priors were used for the mean rate and clock rate parameters. The calibrated Yule model was employed as the tree shape prior. The tree was subjected to secondary calibration for the root of Cricetidae (log-normal distribution with a mean of 18.5 My and standard deviation of 0.85 My) as estimated in ref.^27^. Chain length was 100 million generations, and parameters were logged to a file at every 50,000th step. Convergence diagnostics were performed via Tracer 1.7^28^.

## Results

### Description of the *U. kamensis* karyotype

**Figure 1:**
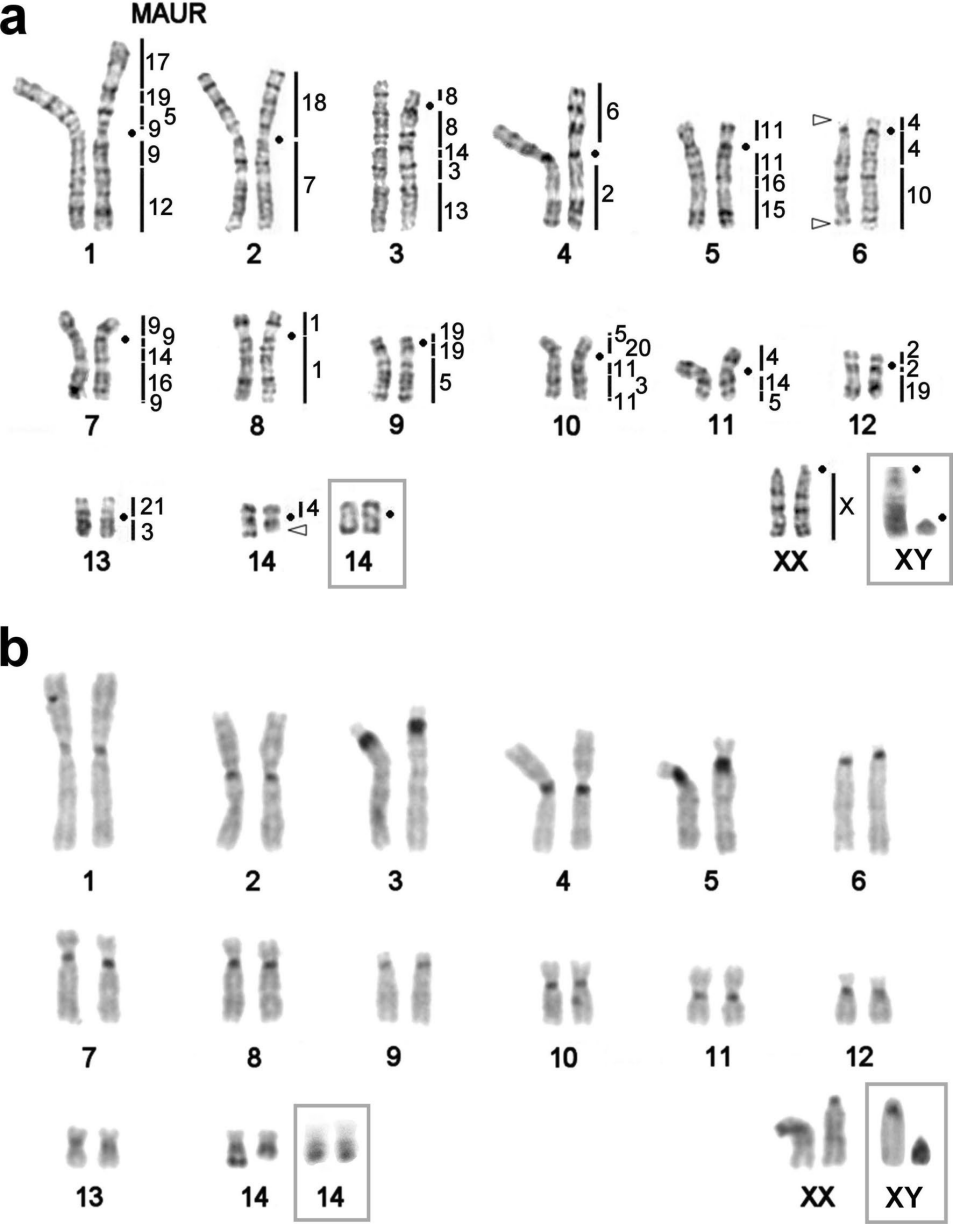
The female karyotype of *U. kamensis*, 2n = 30, NF*a* = 50. (**a**) Localization of *M. auratus* probes (black lines) and the rDNA probe (triangles) on G-banded chromosomes. Centromere positions are marked by black points. (**b**) C-banding. Male sex chromosomes and pair No. 14 are presented in the grey frames.

The diploid chromosome number in karyotypes of the male and female was found to be 2n = 30, NF*a* = 50 (Fig. 1). The autosomal set consists of five pairs of metacentrics (No. 1, 2, 4, 11, and 13), six pairs of submetacentrics (No. 5, 7, 8, 10, and 12), and three pairs of acrocentrics (No. 3, 6, and 9); pair No. 14 turned out to be heteromorphic and is composed of a small meta- and submetacentric. The X chromosome is represented by a medium-size acrocentric. The Y chromosome proved to be the smallest acrocentric in the male karyotype (Fig. 1).

C-banding revealed heterochromatic blocks in pericentromeric regions of all autosomes and X chromosomes (Fig. 1b). The submetacentric homolog of pair No. 14 carries a clearly visible C-positive block in the distal part of the q-arm. The X chromosome contains slightly visible interstitial C-blocks. The Y chromosome was found to be completely C-positive.

**Figure 2:**
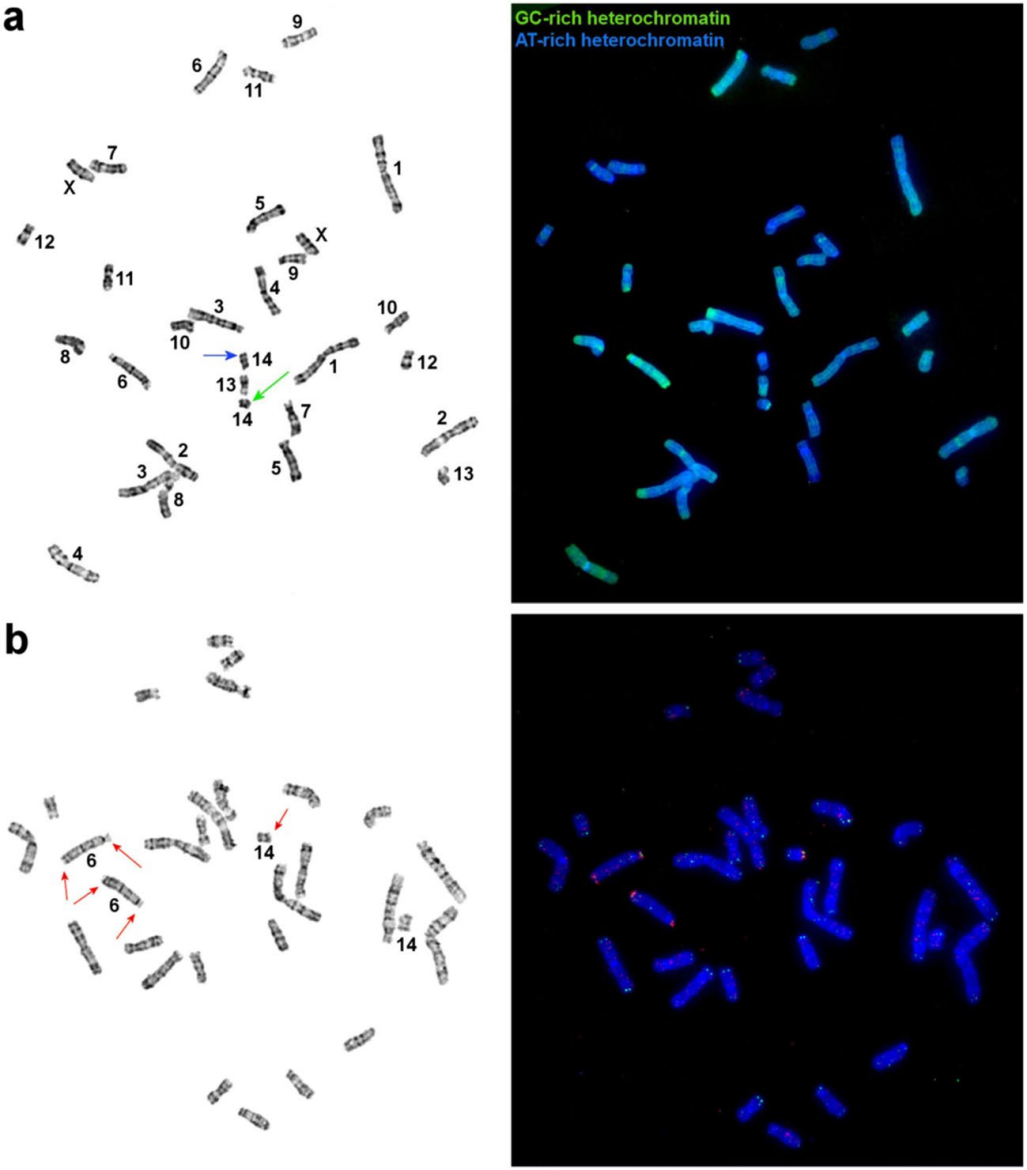
(**a**) CDAG-banding of *U. kamensis* chromosomes; the blue arrow points to a block of AT-rich heterochromatin on one of the homologs of the pair of chromosomes 14, and the green arrow indicates a block of GC-rich heterochromatin on the other homolog of this pair. (**b**) Localization of telomeric (green) and rDNA (red) probes on *U. kamensis* chromosomes; red arrows mark rDNA clusters. G-banded metaphases are shown on the left.

CMA_3_-positive interstitial blocks were seen in chromosomes 2 and X (Fig. 2a). Chromosomes 3 and 6 carry large bright CMA_3_-positive blocks. The smaller homolog of pair No. 14 has a distal CMA_3_-positive region, whereas the bigger one contains a 4′,6-diamidino-2-phenylindole (DAPI)-positive block in the q-arm (Fig. 2a). The hybridization indicated that the smaller homolog carries a cluster of rDNA (Fig. 2b). Two clusters of rDNA were also detected in the p-arm and a distal part of the q-arm of chromosome 6. A telomeric probe did not detect any interstitial localization (Fig. 2b).

### Comparative cytogenetics

The set of chromosome-specific probes of the golden hamster contains several mixed probes^17^. Microdissection was performed to produce individual probes of those chromosomes that hit mixed peaks during flow-sorting. Probes of the following chromosomes of *M. auratus* or their regions were constructed: 6q distal, 6q proximal, 9, 11, 13, 14, 15, and 18 distal.

**Figure 3:**
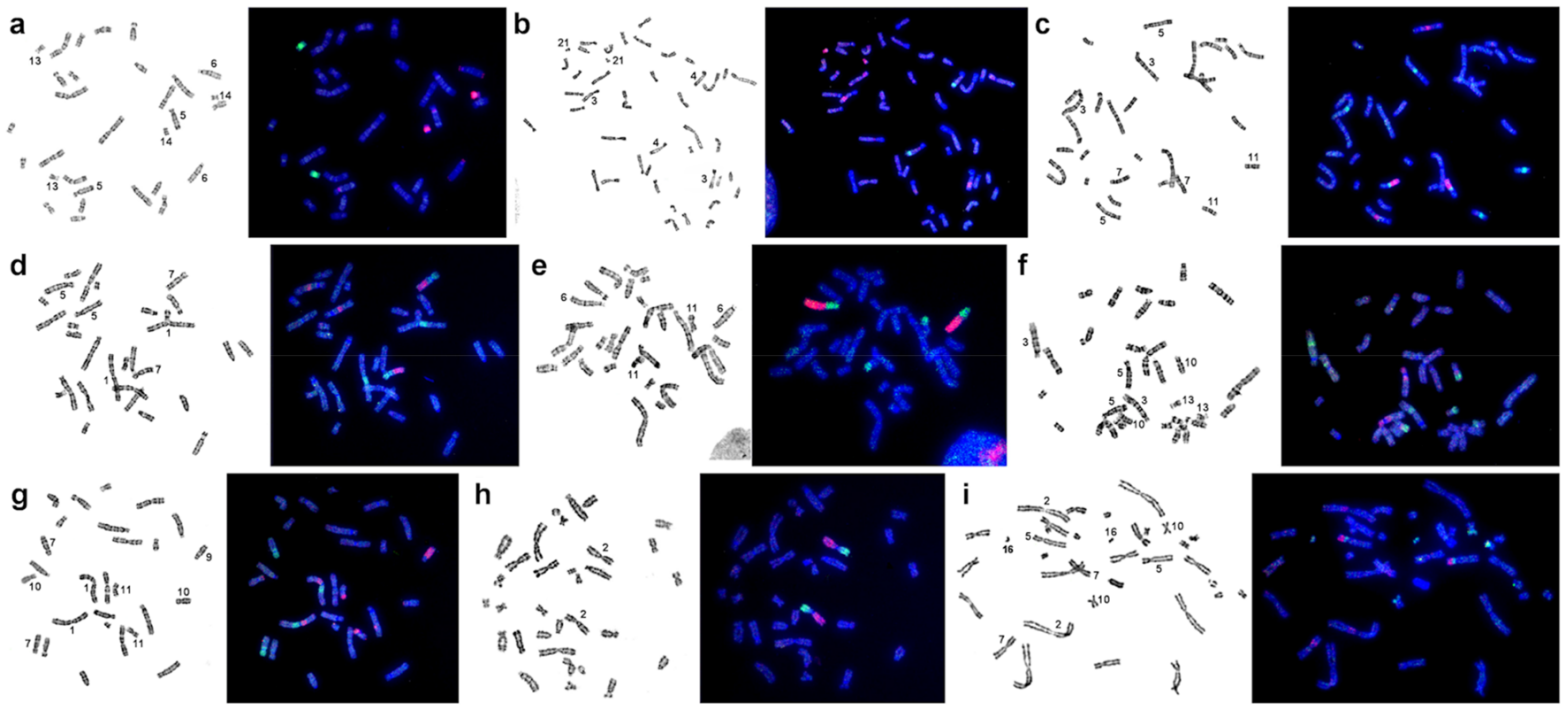
Examples of FISH data. Localization of *U. kamensis* microdissection-derived probes: (**a**), U1 (green) and U2 (red) on chromosomes of *U. kamensis*; (**b**) U3 (green) and U4 (red) on chromosomes of *M. auratus*. Localization of *M. auratus* chromosome–specific probes on chromosomes of *U. kamensis*: (**c**) 14 (green) and 16 (red); (**d**) 9 (green) and 16 (red); (**e**) 4 (green) and 10 (red); (**f**) 3 (green) and 11 (red); (**g**) 9 (green) and 5 (red); and (**h**), 18 (green) and 7 (red). (**i**) Localization of microdissection-derived probes of *M. auratus* 16 (red) and M11 (green) on chromosomes of *P. roborovskii*. G-banded metaphases are presented on the left.

Most probes of *M. auratus* (MAUR), when localized on chromosomes of *U. kamensis*(UKAM), yielded an obvious signal (Fig. 3). No unambiguous signal was detectable on the pair of chromosomes 14 of *U. kamensis*. To determine the region of homology, microdissection of chromosomes 13 and 14 of *U. kamensis* from the same metaphase plate was carried out. Four probes (U1–U4) were obtained. The localization of the resultant probes on chromosomes of the original species and golden hamster was performed next (Fig. 3a,b). It was established that probes U1 and U3 are homologous to chromosome UKAM13 and that U2 and U4 are homologous to chromosome UKAM14 (Fig. 3a). It was demonstrated that the pair of chromosomes 14 of *U. kamensis* shares a homology region with the interstitial region of the q-arm of chromosome 4 of *M. auratus*. A part of chromosome 13 of *U. kamensis* is homologous to the interstitial region of the q-arm of chromosome 3 of *M. auratus* (Figs. 3b). Overall, 38 autosomal conserved segments were uncovered in the *U. kamensis* karyotype owing to the observed localization of the set of chromosome-specific probes of *M. auratus* (Fig. 1a).

**Figure 4:**
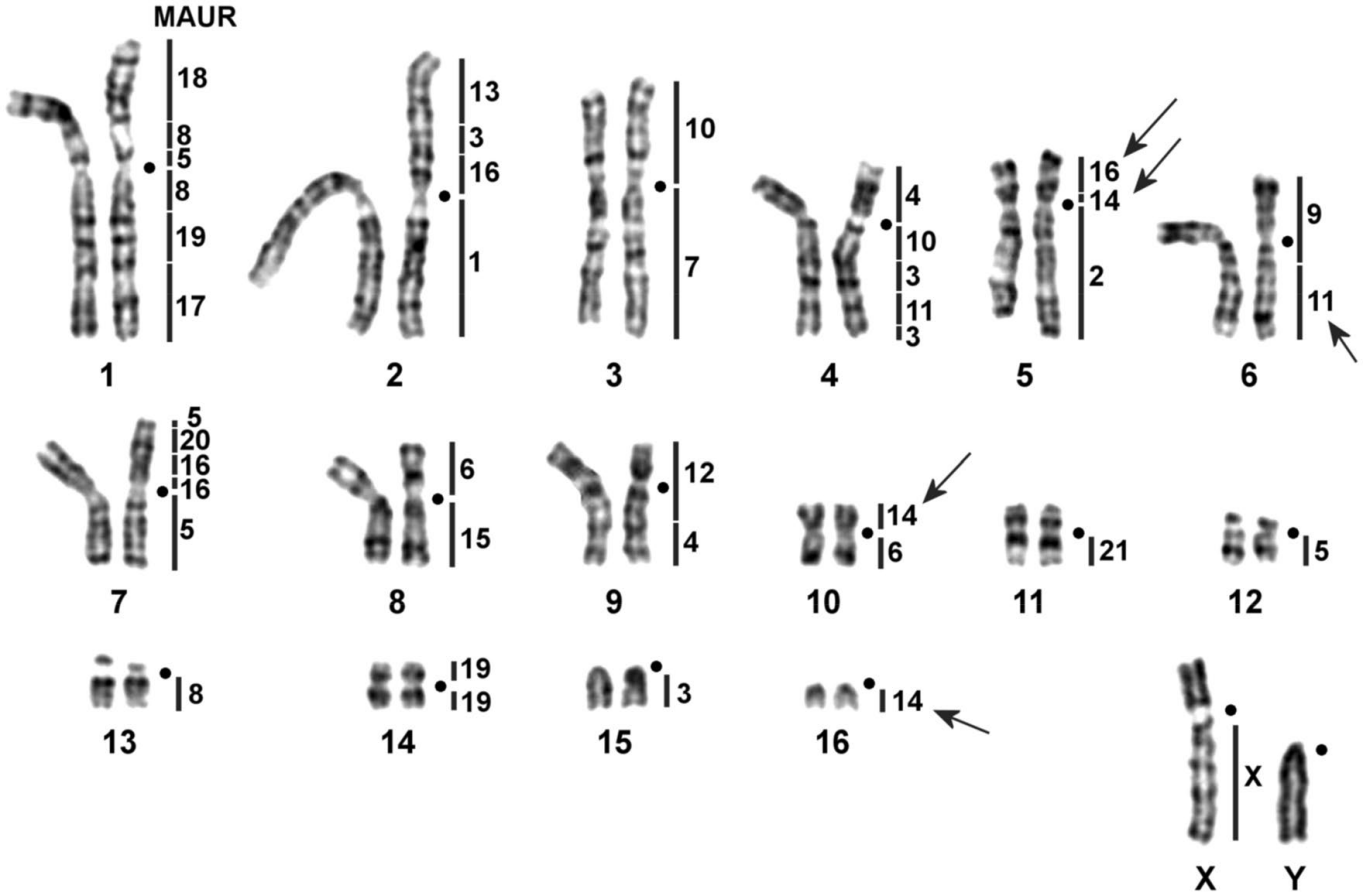
The male karyotype of *P. roborovskii* with corrected localization of *M. auratus* probes (black lines) on G-banded chromosomes. Centromere positions are marked by black points. Black arrows indicate the regions in which the localization of probes changed relative to those published previously^29^.

The new microdissection-derived probes of *M. auratus* and their subsequent localization on chromosomes of two *Phodopus* species (*P. roborovskii* and *P. sungorus*) allowed us to correct the previously published chromosome map for the genus^29^. It was revealed that earlier, in a comparison of banding patterns, the correspondence of the regions homologous to chromosomes MAUR11-14, and 16 was determined incorrectly. We demonstrated that PROB5p is homologous to MAUR16/14 instead of MAUR15, PROB10 is homologous to MAUR6/14 but not MAUR6/11, and PROB16 is homologous to MAUR14 rather than MAUR11 (Fig. 4). Besides, an additional tiny fragment of MAUR5 was uncovered in the distal part of the p-arm of chromosome PROB7. All the detected changes are also characteristic of homologous regions of *P. sungorus* chromosomes (PSUN) and, we believe, *P. campbelli* chromosomes. Microdissection-derived probe UKAM13 hybridized with PSUN5p, PSUN10, PROB11, and PROB15. Probe UKAM14 hybridized with interstitial parts of PSUN1q and PROB4 (data not shown).

### Multilocus phylogenetic trees and molecular dating

The PartititonFinder routine subdivided the alignment of 25 nuclear genes into ten and five subsets for the edge-linked proportional and edge-unlinked models, respectively. The partitioning schemes and optimal substitution models are listed in Supplementary Information. The Bayesian information criterion score was lower for the edge-linked model (76,247.2 versus 76,812.4), consequently indicating a lack of pronounced variation in branch length proportions among the subsets.

**Figure 5:**
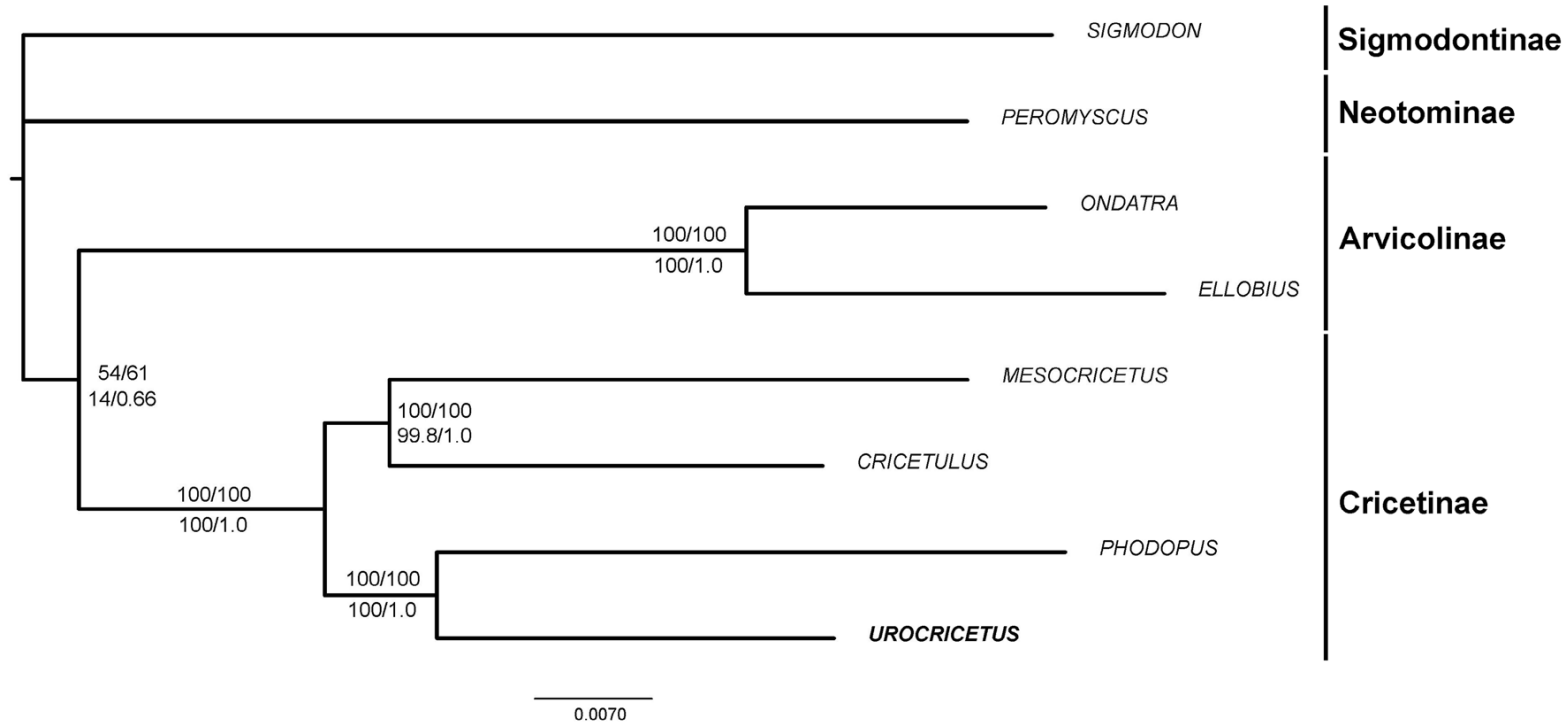
ML phylogeny of Cricetinae as deduced from concatenated alignment of 25 nuclear genes. Clade support is shown at the nodes [top numbers: ML fast bootstrap via edge-linked model (%)/ML fast bootstrap via edge-unlinked model (%); bottom numbers: SH-aLRT support (%)/aBayes support]. Representatives of subfamilies Arvicolinae, Sigmodontinae, and Neotominae serve as outgroups.

Both the ML tree and Bayesian tree (Fig. 5) contained *Phodopus* + *Urocricetus* and *Cricetulus* + *Mesocricetus* clades. This branching pattern is robustly supported by fast bootstrapping, Bayesian posterior probabilities, and P values from the aBayes and SH-aLRT tests. The AU test indicated that the 99.9% credible set contains a single tree.

The gene trees for the 25 markers had various topologies with only 10 genes reproducing the same branching pattern as that in the concatenated tree. The *Phodopus* + *Urocricetus* clade was present in 17 gene trees. Nevertheless, according to the AU tests, none of the gene-specific topologies was significantly better than that of the concatenated tree at P < 0.05. Therefore, all variations among the examined genes can be attributed to insufficient information content in certain alignments rather than to a significant conflict among loci.

According to the node age reconstruction in BEAST (Figure S1), the crown age of Cricetinae dates back to the Middle/Late Miocene boundary (11.2–12.0 Mya). The inferred time of divergence between *Phodopus* and *Urocricetus* (9.2–10.1 Mya) supports an early Late Miocene origin of these lineages.

The obtained two *Cytb* sequences were identical and were found to share the highest similarity with GenBank sequences having accession numbers MK047375, MK047376, and MH142301, differing from them only by seven substitutions. All these sequences belong to clade A as determined in ref.^3^, which we believe to correspond to true *U. kamensis* occurring in eastern Tibet and Nanshan.

## Discussion

*Urocricetus* is one of the few genera of small mammals endemic to Tibet^3^. Numerous studies show that Tibetan fauna species have undoubtedly gone through habitat fragmentation and population fluctuations during glacial cold phases [see reviews in refs.^3, 30^]. It can be assumed that unstable environmental and climatic conditions along with the presence of high-altitude terrains acting as barriers have contributed to rapid chromosomal and molecular evolution.

### Comparative cytogenetics

Karyotype variation within *Urocricetus* remains unstudied. The only previously published piece of information on the chromosome number is the indication that *U. alticola* is characterized by 2n = 22 as reported previously^1^. Nonetheless, the latter publication contains no reference to the original cytogenetic study, and therefore this report needs verification. Here we revealed that *U. kamensis* has 2n = 30, which is higher than 2n in hamsters of the *Cricetulus* group, where 2n = 20–24 is characteristic for all studied species.

The results of comparative chromosome painting convincingly showed that the *U. kamensis* karyotype is very different from that of the rest of cricetine hamsters in terms of the number of detected syntenic blocks and the type of identified *M. auratus* chromosome associations (Fig. 1a). The 38 autosomal conserved segments revealed in the *U. kamensis* karyotype is not only greater than this number in representatives of genera *Cricetulus* and *Phodopus* but also greater than that in all representatives of the subfamily^17, 29^.

In the *U. kamensis* karyotype, we detected some chromosome associations previously identified in karyotypes of other Cricetinae. MAUR17/19 is characteristic for all cricetine hamsters except *Mesocricetus*^29^. On the other hand, in karyotypes of *Cricetulus* and *Allocricetulus*, researchers have registered a full fragment of chromosome MAUR19^17, 29, 31, 32^, whereas in the *U. kamensis* karyotype, the association is formed by a part of MAUR19 (Fig. 1a). Chromosome MAUR19 is represented by three fragments in the genome of this species. Fission of this chromosome is also characteristic of *Phodopus* and *Tscherskia*, where MAUR19 was detected in two fragments [Fig. 4 and Ref.^29^].

In karyotypes of all hamsters except *Mesocricetus*, association MAUR3/13 containing a complete fragment of chromosome 3 has been reported^17, 29, 31, 32^. By contrast, in *U. kamensis* and *Phodopus* karyotypes, the association includes only one of the three fragments of chromosome 3 identified in the genome of the species under study (Fig. 1a)^29^. A similar situation was observed here with the MAUR3/11 association found in karyotypes of absolutely all cricetine hamsters. It is important to note that in the karyotype of *U. kamensis*, the association manifested itself as MAUR11/3/11, whereas in the *P. roborovskii* karyotype it looks like MAUR3/11/3 (Figs. 1a and 4). The question whether these associations have a common evolutionary origin remains open.

Association MAUR4/10, represented by complete fragments of chromosomes 4 and 10, has been identified in the *Allocricetulus curtatus* karyotype^31^. Of note, it is present in two more cricetine species. In *Phodopus* karyotypes, it contains only a fragment of chromosome 10^29^, but in the karyotype of *U. kamensis*, it features a fragment of chromosome 4 (Figs. 1a and 3e).

All representatives of *Cricetulus* and *Allocricetulus* have association MAUR5/9/14/16/15 in their karyotypes^17, 29, 31, 32^. Some components of this association have been documented in karyotypes of other hamsters, e.g., association MAUR5/9/14 in *Phodopus*^29^. Nevertheless, it has been shown that this association contains other parts of *M. auratus* chromosomes. It is likely that associations MAUR5/9, 9/14/16/9, and 15/16 found in the *U. kamensis* karyotype have independent evolutionary origins. Updating previously published data^29^ allowed us to determine precisely that in the karyotypes of *U. kamensis* and *Phodopus* species, MAUR14 is represented by three parts, whereas the number of MAUR16 fragments differs. Thus, in *U. kamensis*, we identified two fragments homologous to MAUR16, and both *Phodopus* species possess three (Figs. 1a and 4).

The discovery of an additional fragment of MAUR5 in *Phodopus* species led to the identification of a new association in their karyotypes (MAUR5/20). A similar association is typical for the *U. kamensis* karyotype, where it was found in the p-arm of chromosome 10 in our study. Previously, association MAUR5q/20 has also been registered in the karyotype of *Tscherskia triton*^29^, however, it is still unknown whether these associations contain identical fragments of chromosome MAUR5.

Our FISH data show that although some associations are shared by *Urocricetus* with other hamsters, and could, in fact, belong to the karyotype of their common ancestor, there is no unambiguous chromosomal synapomorphy supporting an association of *Urocricetus* with any other cricetine lineage.

### Molecular phylogeny of *Urocricetus*

Molecular data convincingly support the hypothesis that *Urocricetus* is a member of a highly divergent lineage that is a sister group to *Phodopus* thereby corroborating previous results based on a less representative sampling (five nuclear genes^2^). According to our molecular-clock analysis, the four major lineages of Cricetinae (*Phodopus*, *Urocricetus*, *Mesocricetus*, and *Cricetus*:*Tscherskia*: *Cricetulus* clade) diverged in quick succession in the earliest Late Miocene, which apparently matches transition to a colder and more arid climate (middle Miocene transition^33^). Relatively short time intervals corresponding to basal internodes in the cricetine tree (1.3–2.0 My) may account for the lack of chromosomal synapomorphies for the *Phodopus* + *Urocricetus* clade.

Considering the intrageneric relationships, it should be emphasized that—contrary to the interpretation by Ding and Liao^3^ who regard *Urocricetus* as monotypic—the available genetic data (both nuclear and mitochondrial) are consistent with recognition of two species within the genus based on the genetic species concept^34^. Thus, mitochondrial phylogeographic data support the existence of two major clades that can be assigned to traditionally accepted *U. kamensis* and *U. alticola*. These clades are separated by the *Cytb* K2P distance of 6.7%, which falls within the divergence range between indisputable sister species of hamsters: i.e., *Phodopus sungorus/Ph. campbelli* (4.5%^35^), *Mesocricetus auratus/M. raddei* (6.0%^35^), and *Cricetulus barabensis* sensu lato/*C. sokolovi* (8.5%^36^). According to nuclear-gene analysis, the split between *U. kamensis* and *U. alticola* dates back to the end of the Early Pleistocene (940 Kya^2^),which is also suggestive of the species level divergence. Moreover, mitochondrial data from ref.^3^ reveal significant structuring within the *U. alticola* mitochondrial clade that consists of three main subclades supposedly corresponding to three subspecies: *U. a. alticola*, *U. a. lama* (Bonhote, 1905), and *U. a. tibetanus* (Thomas, 1922). At present, no morphological, chromosomal, or nuclear multilocus data are available that can elucidate the interrelationships among these taxa.

## Conclusion

Considering the high level of genetic variation within *Urocricetus* and taking into account that Palearctic hamsters are characterized by a high rate of chromosomal evolution—so that in other cricetine genera, chromosomal data have played a major role in the discovery of cryptic species—one may expect that future studies on karyotypic variation within Tibetan hamsters may provide important insights into the taxonomy and evolutionary history of this genus.

## Supplementary Information

Supplementary Information.
