# Supplementary material for: Karyotypic and molecular evidence supports the endemic Tibetan hamsters as a separate divergent lineage of Cricetinae
Source: Sci Rep. 2021 May 18;11:10557. doi: 10.1038/s41598-021-89890-1 (PMC8131748; doi:10.1038/s41598-021-89890-1)
Supplement: Supplementary file 1 — Supplementary Information. [file 41598_2021_89890_MOESM1_ESM.pdf]

Molecular-phylogenetic analysis: experimental part

The specimen Q18-90 of *Urocricetus kamensis* from Qinghai was analysed for 25 nuclear loci and mitochondrial cytochrome *b* gene (*Cytb*). For phylogenetic analysis 175 sequences of different genes of Cricetinae and Arvicolinae were retrieved from GenBank (Table S1).

Total DNA was extracted from ethanol preserved tissues using standard protocol of proteinase K digestion, phenol-chloroform deproteinization and isopropanol precipitation[1]. We sequenced fragments of 25 nuclear loci (Table S2) and 1140 bp of *Cytb*. The 20 original primers were specially designed for amplification and sequencing nuclear genes in this study and the other 10 primers were used from our previous publication[2]. The information about the primers used for amplification and sequencing of nuclear genes are provided in Table S2. Mitochondrial *Cytb* gene was amplified and sequenced using combination of primers L14729[3] and H15985[4].

Polymerase chain reaction (PCR) usually entailed 35 thermal cycles as follows: 94°C for 3 min, 30 cycles of 94°C for 30 s, 55–60°C depending on the primers for 1 min, 72°C for 1 min and a final extension of 72°C for 6 min. PCR products were visualized on 1% agarose gel and then purified using ammonium acetate/ethanol precipitation. Approximately 10–40 ng of the purified PCR product was used for Sanger sequencing with corresponding primers using BigDye™ Terminator v. 3.1 kit (Applied Biosystems, ThermoFisher Scientific), cycle sequencing products were analyzed on 3100-Avant Genetic Analyzer (Applied Biosystems, ThermoFisher Scientific). New sequences were deposited in GenBank under the Accession numbers MW863312-MW863331 and MZ018044-MZ018045.

Optimum models and partition schemes as inferred with ModelFinder

Edge-linked model

HKY+F+G4: acp5_1 adra2b_1 bche_1 dmp1_1 ghr_1 irbp_1 NHSL1_1 vwf_1,
HKY+F+I: acp5_2 adra2b_2 adrb2_1 atp7a_1 Cmyc_1 cnr1_1 lcat_1 NHSL1_2 rag1_1 rag2_1 rag2_2 thy_2 acox2_1 rogdi2_1,
TVM+F+G4: acp5_3 adrb2_3 Cmyc_3 cnr1_3 dmp1_3 irbp_3 lcat_3 NHSL1_3 PRF_3 vwf_3 acpt_3 rogdi2_3,
K2P: adra2b_3 brca2_1 brca2_2 chy_3,
HKY+F+I: adrb2_2 bche_2 Cmyc_2 cnr1_2 lcat_2 rag1_2 acox2_2 acpt_2 rogdi2_2,
TPM3u+F+G4: ApoB_1 ApoB_2 atp7a_3 enam_1 enam_2 ghr_2 irbp_2 PRF_2 rag2_3 vwf_2 thy_3,
K3Pu+F: ApoB_3 bche_3 brca1_3 brca2_3 enam_3,
K2P: atp7a_2 thy_1 acpt_1,
TPM3+F+G4: brca1_1 brca1_2 dmp1_2 PRF_1 chy_1 chy_2,
K2P+G4: ghr_3 rag1_3 acox2_3;

Edge-unlinked model

HKY+F+G4: acp5_1 acp5_2 adra2b_1 adra2b_2 atp7a_1 Cmyc_1 cnr1_1 dmp1_1 dmp1_2 ghr_1 irbp_1 lcat_1 NHSL1_1 NHSL1_2 rag1_1 rag2_1 vwf_1 thy_2 acox2_1 rogdi2_1,

TVM+F+G4: acp5_3 adrb2_3 Cmyc_3 cnr1_3 dmp1_3 irbp_3 lcat_3 NHSL1_3 PRF_3 vwf_3 acpt_3 rogdi2_3,

K3Pu+F+G4: adra2b_3 ApoB_3 atp7a_3 bche_3 brca1_3 brca2_1 brca2_2 brca2_3 enam_3 ghr_3 rag1_3 rag2_3 thy_3 chy_3,

TIM3+F+G4: adrb2_1 ApoB_1 ApoB_2 bche_1 brca1_1 brca1_2 enam_1 enam_2 ghr_2 irbp_2 PRF_1 PRF_2 vwf_2 acox2_3 chy_1 chy_2,

HKY+F+I: adrb2_2 atp7a_2 bche_2 Cmyc_2 cnr1_2 lcat_2 rag1_2 rag2_2 thy_1 acox2_2 acpt_1 acpt_2 rogdi2_2;

Table S1. Sequences of 25 genes of Cricetinae and Arvicolinae retrieved from GenBank

taxon	*Mesocricetus auratus*	*Cricetulus (barabensis) griseus*	*Phodopus ex gr. sungorus*	*Peromyscus sp.*	*Sigmodon sp.*	*Ondatra zibethica*	*Ellobius talpinus*
IRBP	XM_005077637	XM_003495318	MCBN011438683	XM_006976708	EU635706	PVIU01008887	LOEQ01001095
GHR	XM_021223302	NW_003615594	AF540640	AY294927	AF540641	AY294925	LOEQ01002004
RAG1	NW_004801605	NW_003613649	AY294954	NW_006501249	PVIH01014872	PVIU01000102	LOEQ01000880
vWF	AM000038	NW_003613610	AM000049	NW_006501387	PVIH01003003	PVIU01007885	LOEQ01000305
ACP5	NW_004801687	NW_003613752	MCBN011090803	NW_006501211	PVIH01006689	PVIU01021838	LOEQ01010708
ADRA2B	XM_005068583	JN413823	MCBN011457443	NW_006501332	PVIH01000347	PVIU01003202	LOEQ01001076
ADRB2A	NM_001281948	XM_027402776	MCBN011302936	NW_006501285	PVIH01008008	PVIU01015722	LOEQ01008323
ApoB	XM_005079084	JN414053	MCBN011457950	NW_006501198	PVIH01000734	PVIU01009474	LOEQ01003299
ATP7A	XM_005085950	XM_027432881	MCBN010385154	XM_028886391	PVIH01006094	PVIU01013763	LOEQ01007111
BCHE	XM_005076132	XM_027391787	MCBN011456050	XM_028867195	PVIH01025287	PVIU01010962	LOEQ01003330
BRCA2	XM_005083192	AF344828	MCBN011414582	NW_006501358	PVIH01000989	PVIU01006456	LOEQ01001245
C-myc	AY294985	XM_027431576	AY294984	AY294987	AY241507	AY294983	LOEQ01001897
CNR1	FM162115	XM_027403411	MCBN011420962	XM_028872768	PVIH01002493	PVIU01004414	LOEQ01002601
DMP1	XM_005076371	XM_003515794	MCBN011443965	NW_006501096	PVIH01026867	PVIU01006332	LOEQ01003140
ENAM	XM_013111988	XM_027389235	MCBN011450537	XM_028863880	PVIH01000500	PVIU01007871	LOEQ01006989
LCAT	NW_004801665	NW_003614050	MCBN011461676	XM_016005372	PVIH01002868	PVIU01001027	LOEQ01006413
RAG2	FM162083	XM_003497461	MCBN011294972	XM_028895024	PVIH01013589	PVIU01000102	LOEQ01000880
THY1	NW_004801668	NW_003614007	MCBN011148372	NW_006501182	PVIH01021320	PVIU01003711	LOEQ01020783
ACOX2	NW_004801666	NW_003614849	MCBN011419051	NW_006501835	PVIH01010875	PVIU01000936	LOEQ01003411
ACPT	NW_004801779	NW_003614844	MCBN011447908	NW_006501325	PVIH01021081	PVIU01011672	LOEQ01000613
CHY	NW_004801798	NW_003613875	MCBN011422187	NW_006501413	PVIH01018789	PVIU01002672	LOEQ01006019
NHSL1	NW_004801608	NW_003613627	MCBN011242668	NW_006501322	PVIH01001607	PVIU01001783	LOEQ01000757
**PRF**	NW_004801628	NW_003613759	MCBN011219196	NW_006501061	PVIH01000933	PVIU01000824	LOEQ01002017
**ROGDI2**	NW_004801720	NW_003614230	MCBN011010330	NW_006501272	PVIH01025150	PVIU01001008	LOEQ01008065

Table S2. The primers used for amplification and sequencing of nuclear genes

gene		forward primer		reverse primer		annealing T	product length	alignment length (exons only)	internal primers	
brca1	exon	F50	*****	R1120	*****	56,4	1041	1026	R500	F625
irbp	exon	F11	*****	R22Cr	*****	59,5	942	903		
ghr	exon	GHR_F	*****	GHR_R	*****	56,1	909	861	F485	R556
rag1	exon	rag1_all940F	*****	cm_2370a_R	*****	59,3	1499	1446	cm_1800R	cm_1672F
vwf	exon	vwf_ae_f	*****	vwf_cri_r	*****	58,1	793	747		
acp5	exon & intron	F20a	GTRCTGCTGGGCCTGCAAATCAT	R620a	CAGCGCTTGGAGATCTTGGAGTA	59,2	588	315		
adra2b	exon	F1376a	CAACCCTGGCCTCYCCTCTGTCTT	R1894a	GCARTAGCCRATCCARAAGAAGAAC	60,2	545	462		
adrb2a	exon	F217a	ATCGCTACATTGCTATCACATCG	R750b	TAGCCCAACCAGTTAAGGAGGATG	56,9	559	495		
apob	exon	F9632a	GTGTAAARGCYCAATATAAAAAGAACAGAG	R10260d	CCGTGACAAATGAAGAGGAAGAAAG	54,0	597	540		
atp7a	exon	F25a	CTCATCTGATCACMGCAGAGGAAATAAAG	R632b	CTTCAATTRCTTCTCGCAAGGTTTC	55,2	660	597		
bche	exon	F197a	CAAGGTCTGAAAATGTCTTGGTAAAC	R805b	GGAGCAGCTTCAGTGAGCTTACATT	55,3	640	573		
brca2	exon	F5132a	TGTGTCAGTGAGTCTTCTCTATCCAA	R5735a	AGTATCTGGTTTACTCTTTGTGTTTTG	52,1	627	573		
c-myc	exon	F01_cric	GAGGAAGAACAAGAAGATGAGGAAGAAAT	R535_Cric	GTTYCGAAGCTGTTCGAGTTTGTG	58,5	555	483		
cnr1	exon	F18a	CCCTCAARAATTCCCTCTGACTTCCT	H623a	CCAAGGAGAGGCARCACRGCAAT	59,1	633	561		
dmp1	exon	F312a	GGGACAGAAGCTGATAAGGAAGATGA	R918a	GACTCGCTCCTGCTTTCCTCCTTG	58,8	623	555		
enam	exon	F2020a	ACTTTCCMATATATACTCCAGGTCCTACTAT	R2620a	GTTAGTTTATTCTCTGAGATGTTTCTTTGAC	54,7	637	567		
lcat	exon & intron	F55a	GGRTSGACTGCTGGATTGATAATAC	R610a	CTTCCCATAAGCRGCRTACATCTCCTCT	58,4	560	246		
rag2	exon	F292a	GTCTTGCCAGGAGGAATMTCTGTCTC	R863a	GTGCCCMTCTCCATGAGAACAGTAG	55,9	606	534		
thy	exon & intron	THY_F1b	GCCTAACCATCAACACCACCATCTG	THY_R2c	GGCAACAGGGTAGGAGAAATAAGGA	54,4	612	126		
ACOX2	exon & intron	acox_F	GGGCTGTGHAYCACAAACTCCT	acox_R2c	GCTCAGATGAGCAGATTGCTAAATG	56,6	526	96		
ACPT	exon & intron	acpt_f1b	CTTRCCTGGCAGATCARTGTGTC	acpt_r2a	GAGGACAAGGTCAGGRGGCTGGAT	60,7	571	150		
CHY	exon & intron	CHY_R1	GGTAATGGGAGATTCKGGTGAAG	CHY_F3	GACACTCTGCAAGAGGTGAAGATGAG	56,4	546	168		
NHSL1	exon	nhsl1_F	CAACGGGCAGGTGCTCAACGAGAG	nhsl1_R	ACTTCCTCTCYGGRACCTTGGGCTTG	59,3	596	531		
PRF	exon	prf_F1a	CAARGCCTGYGAGGAGAAGAAGAAAC	prf_R2a	CTCAACTCCTGGCCACCAAAGAA	59,4	522	456		
ROGDI-2	exon & intron	rogdi_F1b	GCTSATGGAYGCYGTGATGCTG	rogdi_R	CACGGTGAGGCASAGCTTGTTGA	59,6	515	114		

***** primer sequence is given in Ref.[2]

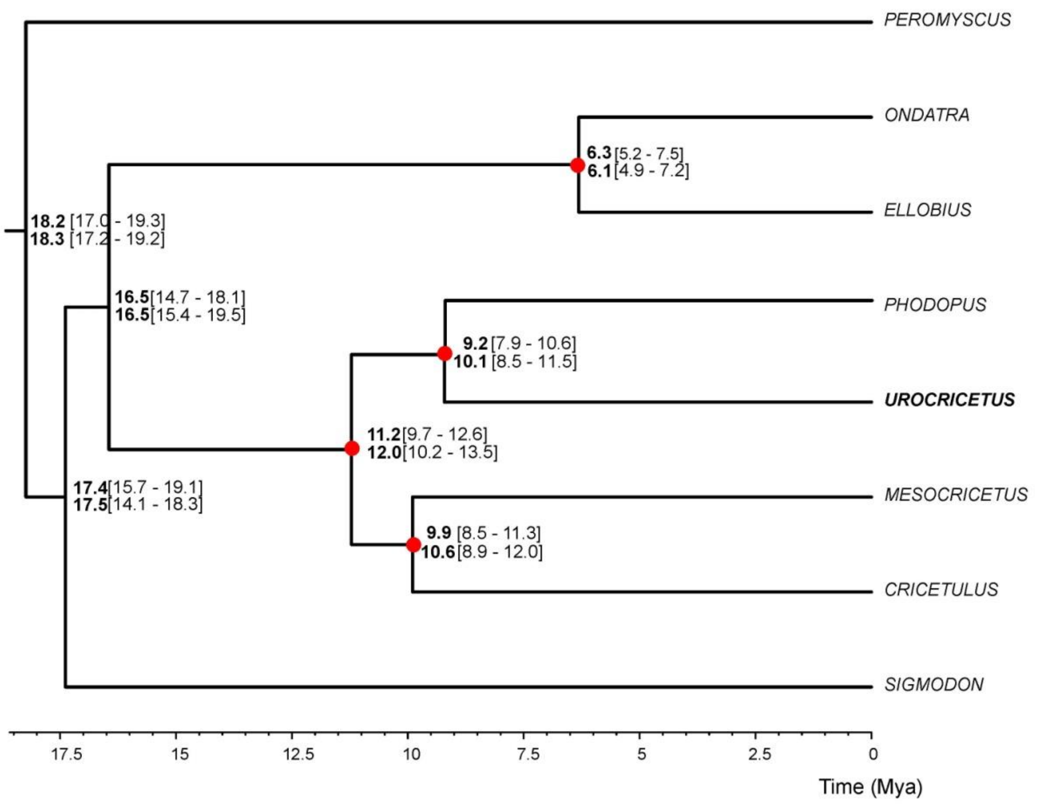

Figure S1. Timescale of major divergence events among Cricetinae taxa as inferred from BEAST analysis under two relaxed-local-clock models based on concatenated alignment of 25 nuclear genes. The median posterior estimate of age (Mya) and 95% height posterior density (HPD) intervals are shown to the right of the nodes (top numbers: the uncorrelated log-normal clock, bottom numbers: the random local clock). The filled red circles denote highly supported nodes in both analyses (Bayesian posterior probabilities >0.99). Representatives of subfamilies Arvicolinae, Sigmodontinae, and Neotominae serve as outgroups.

References

1. Eschbach, S., Hofmann, C., Maerz, M., Maier, U.-G. & Sitte, P. Molecular Cloning. A Laboratory Manual. 2. Auflage. Hrsg. von J. Sambrook, E. F. Fritsch, T. Maniatis, Cold Spring Harbor Laboratory Press, Cold Spring Harbour 1989, $ 115. ISBN 0-87969-309-6. *Biol. unserer Zeit* **20**, 285–285 (1990).
2. Lebedev, V. S. *et al.* Molecular phylogenetics and taxonomy of dwarf hamsters Cricetulus Milne-Edwards, 1867 (Cricetidae, Rodentia): Description of a new genus and

reinstatement of another. *Zootaxa* **4387**, 331–339 (2018).

3. Lebedev, V. S., Bannikova, A. A., Tesakov, A. S. & Abramson, N. I. Molecular phylogeny of the genus Alticola (Cricetidae, Rodentia) as inferred from the sequence of the cytochrome b gene. *Zool. Scr.* **36**, 547–563 (2007).

4. Ohdachi, S., Dokuchaev, N. E., Hasegawa, M. & Masuda, R. Intraspecific phylogeny and geographical variation of six species of northeastern Asiatic Sorex shrews based on the mitochondrial cytochrome b sequences. *Mol. Ecol.* **10**, 2199–2213 (2001).
